# Save the Patella

**DOI:** 10.1051/sicotj/2025004

**Published:** 2025-02-20

**Authors:** Angelo V. Vasiliadis, Vasileios Giovanoulis, Dimitrios Chytas, George Noussios

**Affiliations:** 1 Department of Orthopaedic Surgery, Sports Trauma Unit, St. Luke’s Hospital 55236 Panorama-Thessaloniki Greece; 2 Orthopaedic Surgery and Sports Medicine Department, FIFA Medical Center of Excellence, Croix-Rousse Hospital, Lyon University Hospital Lyon France; 3 Department of Physical Education and Sports Sciences at Serres, Aristotle University of Thessaloniki Greece; 4 Department of Orthopaedic, Centre Hospitalier de Versailles Le Chesnay 78150 France; 5 European University of Cyprus, Engomi Nicosia Cyprus; 6 Basic Sciences Laboratory, Department of Physiotherapy, University of Peloponnese Sparta Greece

Paraphrasing the message “Save the meniscus”, the message “Save the patella” comes to tell us to be more critical on the decision regarding the patella management during total knee arthroplasty (TKA). Patella is not just a bone in the center of the knee joint but it is the largest sesamoid bone in the human skeleton, acting as a fulcrum that increases the lever arm of the extended knee and thereby facilitates the function of the quadriceps muscle [1]. Interestingly, the patella can be characterized as the brain of the knee joint, based on the increased brain activity involved during movement of the knee [2]. Thus, preserving the patella during TKA it is critically important.

Typically, there are three surgical approaches regarding the patella management during TKA: always resurface, never resurface and selectively resurface the patella. However, it seems that the decision to resurface the patella, as part of TKA procedure, is mainly based on the surgeon’s preference and training, geographic location and personal experience [3], rather than evidence-based clinical guidelines. According to different studies and registries, surgeons in the United States routinely resurface the patella (> 90%), while Scandinavian countries, including Norway and Sweden, are on the lower end of the spectrum with only 2% to 3% of the surgeons selecting to resurface the patella [4, 5]. Advocates of routine patellar resurfacing state that it decreases post-operative anterior knee pain, the need for a second operation, thus improving patient-reported outcomes. On the contrary, surgeons who leave the patella unresurfaced, support that this approach minimizes the risk of patellar fracture, avascular necrosis, patellar tendon injury and component loosening [4, 5].

Therefore, the surgeons usually perform either osteophytes removal or/and cartilage smoothing “patelloplasty” in moderate cases [5]. The loss of the cushioning effect of the articular cartilage, in cases of degeneration of the patellar cartilage, leads the subchondral bone experiences increased pressure, while the underlying nerves were exposed, resulting in pain and functional impairment of the affected joint [6]. In these cases, patellar denervation may help to decrease post-operative anterior knee pain, improve patient’s satisfaction and minimize the complications [6]. Furthermore, in severe cases with extensor mechanism tightness, partial lateral patella facetectomy or lateral retinaculum release were proposed to restore central patellar tracking and improve knee kinematics (Figure 1) [7]. However, the choice to resurface the patella during TKA is a combination of the above-mentioned factors (Figure 2) with the addition to this “equation” of the thickness, the shape and the osteometabolic state of the patella [8]. Among the contraindications to patellar resurfacing is a thin and eroded patella, where the thickness after resection would be less than 12 mm [4]. In addition, poor bone quality of the patella can ultimately lead to poor bone-prosthesis or bone-cement-prosthesis integration and to an increased risk for periprosthetic fracture, making the patella unsuitable for a resurfacing procedure (Figure 2) [8].

**Figure 1:**
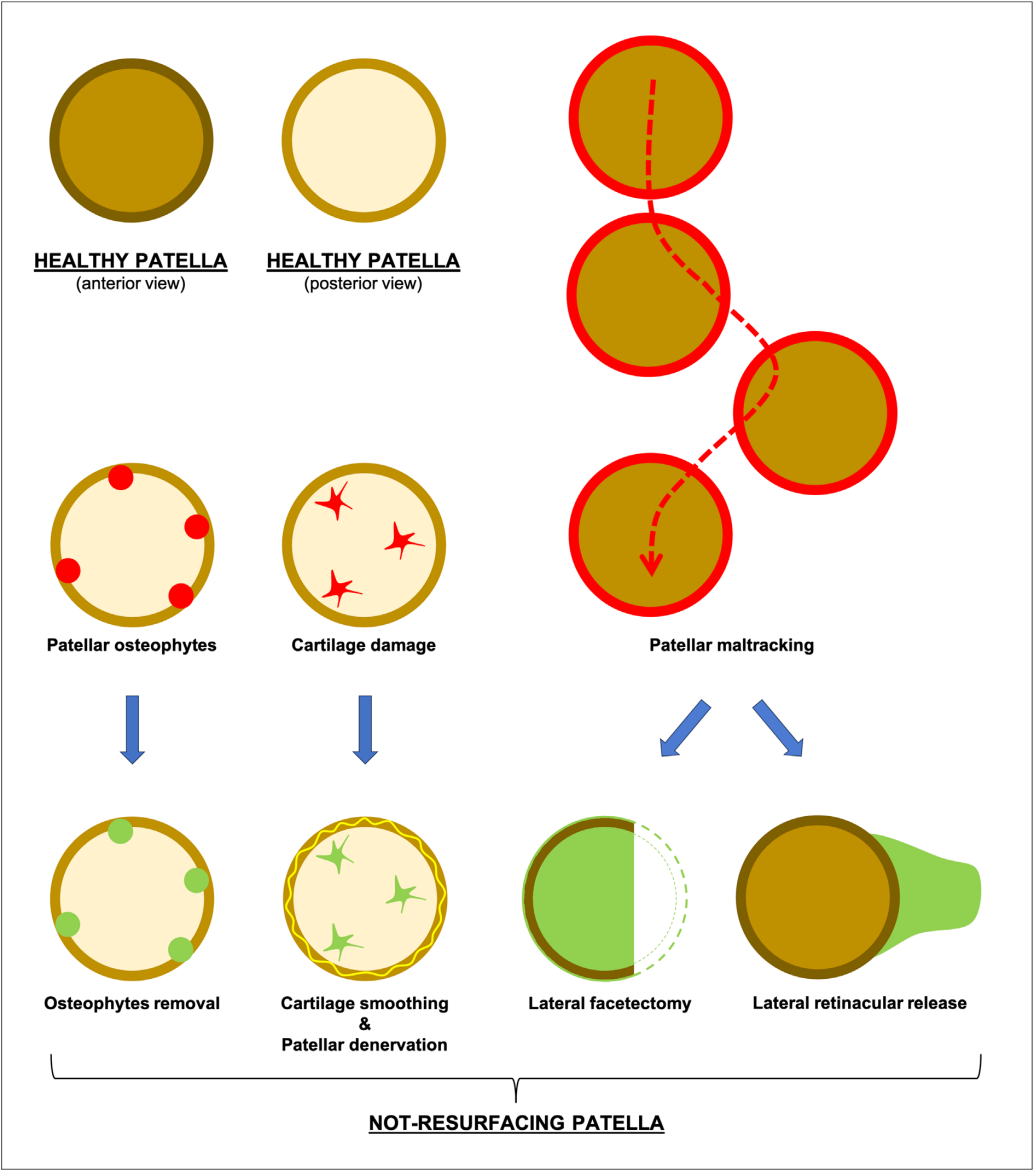
Illustration shows the patella management during total knee arthroplasty (the option of not-resurfacing the patella).

**Figure 2:**
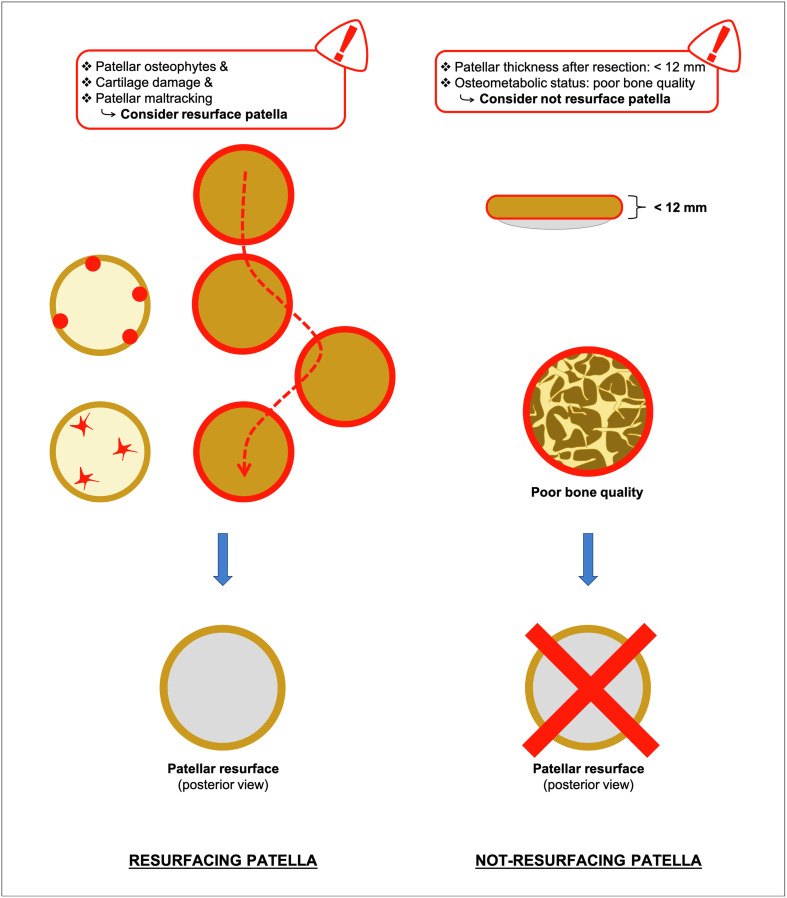
Illustration shows the patella management during total knee arthroplasty (the option of resurfacing the patella and the factors to take into consideration).

In 2024, we may continue to debate over the patellar management during primary TKA, but we can all agree that up to 20% of patients undergoing TKA remain unsatisfied, regardless of resurfacing of the patella [4, 9]. Despite the substantial advances in primary TKA regarding the surgical technique, instrumentation, alignment strategy and implant design, patient satisfaction is a relative concept that is strongly linked to patient expectations. Several studies have been conducted to investigate the satisfaction rate of secondary patellar resurfacing, demonstrating that the overall dissatisfaction rate ranged from 41% to 65% [10–14]. Toro-Ibarguen et al., in a retrospective study, showed that in 59% of the patients who underwent secondary patellar resurfacing for persistent anterior knee pain, there was no pain improvement, while 65% of the patients were dissatisfied [10]. The authors concluded to be less likely to recommend this procedure as a treatment option for this subgroup of patients. More recently, Thomas et al. demonstrated that 66% of patients did not benefit from secondary patella resurfacing and emphasized the importance of clarifying the cause of anterior knee pain after the primary TKA [11]. These findings are also supported by a systematic review and meta-analysis including six studies of 604 knees, with a mean follow-up period of 42 months [12]. The authors demonstrated that 41% of the patients were dissatisfied after secondary patella resurfacing with half of the patients not achieving improvements in anterior knee pain and functional ability. A complication rate ranging between 3.4% and 30% among the included studies was noted [12]. In conclusion, despite the trend towards higher re-operation rates with unresurfaced patella, resurfacing the patella during primary TKA may not be necessary, due to the fact that it is not clear that the need of re-operation is a direct consequence the patella being unresurfaced [13]. Finally, the increase of costs and health-care resources during TKA, with patella resurfacing, needs to be considered during the decision-making process [14].

Of course, the moto “Save the Patella” does not mean “Do not Resurface the Patella”, but proceed with caution following the indications for patellar resurfacing. Therefore, it is crucial to develop accurate criteria to identify patients would benefit from primary patella resurfacing. Also, within patients with persistent anterior knee pain, who had not undergone patella resurface, it is critical to identify the best candidates for secondary patella resurfacing. In this aspect, malrotation and aseptic or septic loosening of TKA components represent exclusion criteria. Maybe something less personal like. Any senior or junior surgeon needs to remember this moto:

*Save the Patella*.

## Data Availability

Data are available on request from the authors.

## References

[1] Konrads C, Schreiner AJ, Cober S, Schüll D, Ahmad SS, Alshrouf MA (2022) Evaluation of patella height in native knees and arthroplasty: an instructional review. SICOT J 8, 36. 35997518 10.1051/sicotj/2022037 PMC9397114

[2] Callaghan MJ, McKie S, Richardson P, Oldham JA (2012) Effects of patellar taping on brain activity during knee joint proprioception tests using functional magnetic resonance imaging. Phys Ther 92, 821–830. 22282771 10.2522/ptj.20110209 PMC3367140

[3] Chithartha K, Nair AS, Thilak J (2021) A long-term cross-sectional study with modified forgotten joint score to assess the perception of artificial joint after total knee arthroplasty. SICOT J 7, 14. 33704059 10.1051/sicotj/2021013 PMC7949890

[4] Abdel M, Parratte S, Budhiparama NC (2014) The patella in total knee arthroplasty: to resurface or not is the question. Curr Rev Musculoskelet Med 7, 117–124. 24706154 10.1007/s12178-014-9212-4 PMC4092199

[5] McConaghy K, Derr T, Molloy RM, Klika AK, Kurtz S, Piuzzi NS (2021) Patellar management during total knee arthroplasty: A review. EFORT Open Rev 6, 861–871. 34760286 10.1302/2058-5241.6.200156 PMC8559560

[6] Nkachukwu K, Alejo A, Toman J, Jwayyed J, Iwuagwu J, Alejo A (2024) Denervation of the patella during knee arthroplasty: An updated systematic global review. J Clin Med 13, 6942. 39598085 10.3390/jcm13226942 PMC11594293

[7] Ferri R, Digennaro V, Panciera A, Bulzacki Bogucki BD, Cecchin D, Manzetti M, Brunello M, Faldini C (2022) Management of patella maltracking after total knee arthroplasty: a systematic review. Musculoskelet Surg 107, 143–157. 36197592 10.1007/s12306-022-00764-9 PMC10191954

[8] Molfetta L, Casabella A, Palermo A (2021) The patellar resurfacing in total knee prosthesis: Indications for bone stock and patellar morphology. Front Med (Lausanne) 7, 405. 33718393 10.3389/fmed.2020.00405 PMC7943458

[9] Kafelov M, Farhat J, Servien E, Lustig S, Batailler C (2023) New measurement technique for restoration of the trochlear offset after image-based robotic-assisted total knee arthroplasty: a reliability study. SICOT J 9, 29. 37772857 10.1051/sicotj/2023027 PMC10540675

[10] Toro-Ibarguen AN, Navarro-Arribas R, Pretell-Mazzini J, Prada-Canizares AC, Jara-Sanchez F (2016) Secondary patellar resurfacing as a rescue procedure for persistent anterior knee pain after primary total knee arthroplasty: Do our patients really improve? J Arthroplasty 31, 1539–1543. 27038861 10.1016/j.arth.2016.01.001

[11] Thomas C, Patel V, Mallick E, Esler C, Ashford RU (2018) The outcomes of secondary resurfacing of the patella following total knee arthroplasty: Results from the Trent and Wales Arthroplasty Register. Knee 25, 146–152. 29366665 10.1016/j.knee.2017.10.004

[12] Andronic O, Suravaram V, Lu V, Wall SJ, Bucher TA, Prosser GH, Yates PJ, Jones CW (2024) What are the outcomes of secondary patella resurfacing for dissatisfaction following primary knee arthroplasty? A systematic review and meta-analysis of 604 knees. J Arthroplasty 39, 1093–1107. 37871862 10.1016/j.arth.2023.10.027

[13] Delgado-González A, Morales-Viaji JJ, Arteaga-Hernández JG, Larrosa-Arranz A, Criado-Albillos G, Martin-Rodriguez AP, Jahouh M, González-Santos J, Mendieta Diaz L, Collazo Riobo C, Calvo Simal S, González-Bernal JJ (2022) To resurface or not to resurface the patella in total knee arthroplasty, that is the question: A meta-analysis of randomized controlled trials. Medicina (Kaunas) 58, 227. 35208551 10.3390/medicina58020227 PMC8875724

[14] Held MB, Gazgalis A, Sarpong NO, Geller JA, Shah RP Management of the patella during total knee arthroplasty. JBJS Rev 9:10.2106/JBJS.RVW.21.00054. 34516451

